# Utilisation of *Lactiplantibacillus plantarum* and propionic acid to improve silage quality of amaranth before and after wilting: fermentation quality, microbial communities, and their metabolic pathway

**DOI:** 10.3389/fmicb.2024.1415290

**Published:** 2024-06-06

**Authors:** Muqier Zhao, Jian Bao, Zhijun Wang, Pengbo Sun, Jingyi Liu, Yuting Yan, Gentu Ge

**Affiliations:** ^1^College of Grassland, Resources and Environment, Inner Mongolia Agricultural University, Hohhot, China; ^2^Key Laboratory of Forage Cultivation, Processing and High Efficient Utilization of Ministry of Agriculture and Rural Affairs, Inner Mongolia Agricultural University, Hohhot, China; ^3^Inner Mongolia Academy of Agricultural and Animal Husbandry Sciences, Hohhot, China

**Keywords:** amaranth silage, fermentation quality, *Lactiplantibacillus plantarum*, microbial community, moisture content, propionic acid

## Abstract

**Objective:**

The aim of this study was to investigate the effects of *Lactiplantibacillus plantarum* (*L. plantarum*) and propionic acid (PA) on fermentation characteristics and microbial community of amaranth (*Amaranthus hypochondriaus*) silage with different moisture contents.

**Methods:**

Amaranth was harvested at maturity stage and prepared for ensiling. There were two moisture content gradients (80%: AhG, 70%: AhS; fresh material: FM) and three treatments (control: CK, *L. plantarum*: LP, propionic acid: PA) set up, and silages were opened after 60 d of ensiling.

**Results:**

The results showed that the addition of *L. plantarum* and PA increased lactic acid (LA) content and decreased pH of amaranth after fermentation. In particular, the addition of PA significantly increased crude protein content (*p* < 0.05). LA content was higher in wilted silage than in high-moisture silage, and it was higher with the addition of *L. plantarum* and PA (*p* < 0.05). The dominant species of AhGLP, AhSCK, AhSLP and AhSPA were mainly *L. plantarum*, *Lentilactobacillus buchneri* and *Levilactobacillus brevis*. The dominant species in AhGCK include *Enterobacter cloacae*, and *Xanthomonas oryzae* was dominated in AhGPA, which affected fermentation quality. *L. plantarum* and PA acted synergistically after ensiling to accelerate the succession of dominant species from gram-negative to gram-positive bacteria, forming a symbiotic microbial network centred on lactic acid bacteria. Both wilting and additive silage preparation methods increased the degree of dominance of global and overview maps and carbohydrate metabolism, and decreased the degree of dominance of amino acid metabolism categories.

**Conclusion:**

In conclusion, the addition of *L. plantarum* to silage can effectively improve the fermentation characteristics of amaranth, increase the diversity of bacterial communities, and regulate the microbial community and its functional metabolic pathways to achieve the desired fermentation effect.

## Introduction

As the global economy continues to grow and the population continues to increase, so does the demand for animal products, such as meat, eggs and milk (Flachowsky, 2017). In terms of livestock production, many countries are dependent on imported feed due to the increased demand for forage, which has resulted in forage production not being able to meet the demand for animal feed. Therefore, the development of new forage resources, such as nutritious amaranth, is urgently needed to solve the problem of feed shortage brought about by the rapid development of animal husbandry (Du et al., 2022). Amaranth (*Amaranthus hypochondriaus*) is an annual plant that has been cultivated for 8,000 years and may be a potential source of protein feed (Rezaei et al., 2014; Peiretti, 2018). Amaranth is a source of high-quality proteins such as albumin and globulin, and is rich in lysine and methionine, which are two amino acids lacking in other grains. It also shows very high bioavailability, which explains the high protein content (about 14%DM) of amaranth, hence the potential to be a substitute for animal foods (Rivero Meza et al., 2022).

The moisture content of the raw material, ensiling time and the use of additives are key factors affecting the quality of the fermentation. When moisture exceeds 70%, it can lead to clostridial fermentation. Wilting is widely used in forage fermentation preparation mainly by regulating forage moisture to reduce microbial activity in water and inhibit the proliferation of microorganisms that are harmful to fermentation, such as *Clostridium* (Kung et al., 2018). To improve the silage process (e.g., rate of acid formation, aerobic stability), fermentation enhancers and inhibitors may be added (Benjamim da Silva et al., 2022; Li et al., 2023), such as chemicals, starter cultures, or lactic acid bacteria (LAB), which will result in a rapid decrease in pH. The typical fermentation enhancers are LAB-based inoculants, which are classified as homofermentative (producing only LA) and heterofermentative (producing both LA and acetic acid) (Muck et al., 2018). Supplementation of inoculants alters the microbial community structure and fermentation products in silage (Benjamim da Silva et al., 2022; Drouin et al., 2022). Typical fermentation inhibitors are mixtures of organic acids, such as formic and propionic acid (PA), which directly lower the pH of the forage, thus preventing the consumption of carbohydrates by the fermentation process (Kung et al., 2003). Organic acid addition also inhibits the multiplication of spoilage microorganisms in silage (Muck et al., 2018).

Fresh amaranth was characterized by high moisture content and low water-soluble carbohydrate (WSC) content (about 2%DM). Conventional production methods, hay, may increase lignin content, resulting in amaranth leaf abscission (Zhao et al., 2022). Furthermore, amaranth has a thick main stem axis and high moisture content (Das, 2016), which is difficult to be dried quickly and has relatively high mold losses, resulting in its inability to produce the hay in the rainy season, whereas making it into silage allows for long term preservation while reducing the loss of nutrients. However, amaranth is high in moisture content and sugar content, resulting in lower quality of direct silage (Rezaei et al., 2009). Therefore, ensiling is considered as an ideal preparation and storage method to solve problems (Murphy, 2011). Ensiling is a traditional, important and reliable technology for preserving forage and crops. The fermentation process plays a crucial role in livestock development mainly through the microbial decomposition of WSC to produce organic acids dominated by lactic acid (LA) (Muck et al., 2018; Ren et al., 2020), which results in the formation of an acidic anaerobic environment and inhibits the growth of spoilage microorganisms (Zhao et al., 2023).

Fermentation is a dynamic process of microbial community succession and metabolite changes (Ding et al., 2013). Third-generation PacBio single-molecule real-time (SMRT) sequencing technology can detect species-level sequencing reads in complex anaerobic fermentation ecosystems to modulate bacterial community succession as well as bacterial interactions and metabolic pathways (Amir et al., 2013). In order to explore the characteristics of amaranth as a new forage resource that can change the mode of agricultural and livestock production, the present study set up two moisture content gradients for amaranth with high moisture content, regulated the fermentation characteristics and bacterial community diversity of amaranth silage through the addition of *Lactobacillus plantarum* (*L. plantarum*) and propionic acid (PA), and explored the structure of microbial community, its functional characteristics and anaerobic fermentation mechanism of amaranth silage by utilizing PacBio SMRT sequencing technology.

## Methods

### Experimental design and silage preparation

The experimental material was red-fruited amaranth, which was sown on 9 June 2021 at the pasture experimental base of Inner Mongolia Agricultural University (111°430 E, 40°480 N). Six 1 m^2^ plots were selected for harvesting on 10 August 2021 (maturity stage). The harvested amaranth was placed on clean plastic sheets and when the moisture content was around 70 and 80%, the amaranth was guillotined into lengths of about 2–3 cm using a hand-held guillotine for silage making.

The prepared amaranth with different moisture contents were divided into six groups: (1) no additive control (labelled as AhGCK and AhSCK). (2) *L. plantarum* JYLP-002, was added at a ratio of 1 × 10^6^ cfu *L. plantarum*/g of fresh matter (FM), labelled as AhGLP and AhSLP (supplied by Shandong Zhongke Jiayi Bioengineering Co., Ltd., China). (3) PA was added at a 4 g/kg ratio of FM, labelled as AhGPA and AhSPA (supplied by Shanghai McLean Biochemical Technology Co.). Both additives were dissolved in deionised water and 10 mL of solution per kg of amaranth was sprayed with a hand sprayer. The control silage was sprayed with an equal amount of deionised water. Amaranth (500 g) was packed in polyethylene bags, then vacuum-sealed for 60 d of ensling for measurement.

### Chemical and fermentation characteristics analysis

There were six replicates for each amaranth silage sample. Dry matter (DM) and crude protein (CP) content were determined according to Zhang et al. (2016) and Patrica (1997), respectively. Neutral detergent fiber (NDF) and acid detergent fiber (ADF) contents were determined according to Van Soest et al. (1991). WSC content was measured according to Chen et al. (2015).

Silage samples (10 g) were mixed with 90 g of deionised water as described by Cai (2004) and stored in a refrigerator at 4°C for 1 d. Then, the leachate was filtered through four layers of gauze and filter paper for determination of pH, ammonia nitrogen (NH_3_-N) and organic acids in the leachate. pH was measured using a glass electrode pH meter (Mettler Toledo; model: FE28, Shanghai, China). Organic acid (LA, acetic acid: AA, and PA) in silage was determined by high performance liquid chromatography (HPLC; model: Waters e2695, Milford, United States) (Cheng et al., 2020). NH_3_-N concentrations were determined using the method of Broderick and Kang (1980). Microbial populations (LAB, yeast, molds, anaerobic bacteria and *Escherichia coli*) in FM were assessed according to a previous report (You et al., 2021).

### Microbial community analysis

Bacterial community composition of fermented 60 d of amaranth was analysed by 16S rRNA gene sequencing. Total DNA was extracted from fresh and silage amaranth samples according to Liu et al. (2019), and metagenomic DNA extraction and PCR amplification of bacterial 16S rRNA gene were performed according to Guo et al. (2021) with primers 27F (5’-AGRGTTTGATYNTGGCTCAG-3′) and 1492R (5’-TASGGHTACCTTGTTASGACTT-3′) and PCR conditions were according to the study of Zhao et al. (2023). The PCR products were purified for sequencing and analysis. Each treatment was performed six times. 16S rRNA gene sequences were stored in the Biological Programmes at NCBI under accession number PRJNA1049970.

NGS sequencing was performed by Biomarker Technologies (Beijing, China) on the Pacbio_SMRT platform (Pacbio Sequel II, California, United States). Principal component analysis (PCA) of β-diversity based on unweighted or weighted unifrac distances was plotted using the R program (version 3.2.5). The SILVA (version 128) 16S rRNA database was classified by Operational Taxonomic Unit (OTU) using the Ribosomal Database Project (RDP) classifier (version 2.2) with a minimum confidence of 0.7, and then by phylum, genus, and species. The alpha diversity indices (ACE, Chao 1, Simpson and Shannon) of the samples were assessed using Mothur (version v.1.30) software (Caporaso et al., 2010), and microbial functions in the Kyoto Encyclopaedia of Genomes (KEGG) database were predicted using PICRUSt2 software. All figures were produced using the free platform.^1^

## Results

The chemical and microbial composition of pre-ensiled amaranth are shown in Table 1. The DM content of AhSFM was significantly higher than that of AhGFM (*p* < 0.05), and the CP, NDF, ADF, and WSC content of AhSFM was higher than that of AhGFM, and the number of LAB, yeast, and *Escherichia coli* was higher than AhGFM (*p* > 0.05). However, aerobic bacteria (*p* < 0.05) and molds (*p* > 0.05) were higher in AhGFM than in AhSFM.

**Table 1:** Chemical and microbial composition of pre-ensiled amaranth.

Items	AhGFM (80%)	AhSFM (70%)
DM (%FM)	21.00 ± 1.23b	32.03 ± 0.16a
CP (%DM)	6.94 ± 0.06a	7.99 ± 0.85a
NDF (%DM)	46.37 ± 2.68a	47.39 ± 0.93a
ADF (%DM)	33.22 ± 1.05a	36.70 ± 0.74a
WSC (%DM)	1.18 ± 0.05a	1.35 ± 0.28a
Lactic acid bacteria (Log_10_ cfu/g FM)	3.84 ± 0.55a	3.90 ± 0.78a
Coliform bacteria (Log_10_ cfu/g FM)	2.73 ± 2.53a	4.65 ± 2.04a
Aerobic bacteria (Log_10_ cfu/g FM)	6.45 ± 0.73a	4.03 ± 2.15b
Yeast (Log_10_ cfu/g FM)	4.55 ± 2.58a	5.26 ± 0.37a
Molds (Log_10_ cfu/g FM)	2.00 ± 1.76a	0.90 ± 0.39a

FM, fresh matter; DM, dry matter; CP, crude protein; NDF, neutral detergent fiber; ADF, acid detergent fiber; WSC, water-soluble carbohydrate.

The effects of different moisture contents and additives on the nutrient content of amaranth silage are shown in Table 2. Moisture content had a highly significant (*p* < 0.01) effect on DM and CP content. However, additives had no significant effect (*p* > 0.05) on the nutrient indexes. There was a highly significant (*p* < 0.01) interaction between the two factors on CP content. Both AhGPA and AhSPA had significantly higher CP content than other groups (*p* < 0.05). The DM content of AhSCK, AhSLP and AhSPA was significantly (*p* < 0.05) higher than AhGCK, AhGLP and AhGPA. The CP content of AhSCK and AhSLP was significantly (*p* < 0.05) higher than AhGCK and AhGLP, while AhSPA was significantly (*p* < 0.05) lower than AhGPA. None of other nutrient indices were significantly different (*p* > 0.05) at different moisture content gradients.

**Table 2:** Chemical compositions on 60 days of ensiling.

Items	80%			70%			SEM	Significance		
	AhGCK	AhGLP	AhGPA	AhSCK	AhSLP	AhSPA		Moisture	Additive	M × A
DM(%FM)	20.90 ± 1.39Ab	21.13 ± 0.16Ab	21.03 ± 0.59Ab	31.46 ± 0.72Aa	31.37 ± 0.51Aa	31.01 ± 0.29Aa	2.30	<0.0001	0.8887	0.8387
CP (%DM)	5.90 ± 0.69Bb	6.15 ± 0.26Bb	8.08 ± 0.12Aa	7.92 ± 0.85ABa	8.59 ± 0.75Aa	6.35 ± 0.98Bb	0.47	0.0092	0.4987	0.0002
NDF (%DM)	48.31 ± 2.19Aa	44.83 ± 2.69Aa	46.38 ± 2.41Aa	46.44 ± 1.94Aa	46.17 ± 0.74Aa	46.53 ± 0.89Aa	0.45	0.2392	0.1098	0.1041
ADF (%DM)	39.37 ± 1.18Aa	36.09 ± 1.58Aa	38.07 ± 2.15Aa	39.23 ± 1.29Aa	38.52 ± 1.59Aa	42.10 ± 1.65Aa	0.80	0.1060	0.2057	0.3267
WSC (%DM)	0.37 ± 0.10Aa	0.31 ± 0.09Aa	0.23 ± 0.05Aa	0.16 ± 0.03Aa	0.25 ± 0.06Aa	0.32 ± 0.13Aa	0.03	0.2946	0.1305	0.9866
pH	5.09 ± 0.39Aa	4.16 ± 0.05Bb	4.28 ± 0.12Ba	4.51 ± 0.04Aa	4.20 ± 0.01Ba	4.17 ± 0.04Ca	0.15	0.0242	<0.0001	0.0114
LA(%FM)	1.42 ± 0.20Cb	3.65 ± 0.12Ab	2.52 ± 0.28Bb	2.85 ± 0.03Ba	4.46 ± 0.09Aa	3.53 ± 0.08Aa	0.47	<0.0001	<0.0001	<0.0001
AA(%FM)	0.67 ± 0.10Aa	0.40 ± 0.09Ba	0.17 ± 0.04Cb	0.21 ± 0.03Bb	0.19 ± 0.04Bb	0.59 ± 0.09Aa	0.45	0.0143	0.0051	<0.0001
PA (%FM)	0.35 ± 0.13Ba	0.12 ± 0.02Ca	0.98 ± 0.13Aa	0.23 ± 0.14Ba	0.18 ± 0.06Ba	0.82 ± 0.10Aa	0.15	0.1456	<0.0001	0.0114
NH_3_-N/TN	1.42 ± 0.32Aa	1.02 ± 0.03Aa	1.22 ± 0.10Aa	1.17 ± 0.08Aa	1.04 ± 0.22Aa	1.06 ± 0.08Aa	0.06	0.1357	0.0789	0.4556

FM, fresh matter; DM, dry matter; CP, crude protein; NDF, neutral detergent fiber; ADF, acid detergent fiber; WSC, water-soluble carbohydrate. Different capital letters indicate significant differences between additive treatments at the same moisture content gradient (*p* < 0.05), the same capital letters indicate non-significant differences (*p* > 0.05). Different lower case letters indicate significant (*p* < 0.05) differences in the same treatment across moisture content gradients, the same lower case letters indicate non-significant (*p* > 0.05) differences. LA, lactic acid; AA, acetic acid; PA, propionic acid; NH_3_-N, ammonia nitrogen; TN, total nitrogen.

There was a highly significant (*p* < 0.01) effect of moisture content on LA content and significant (*p* < 0.05) effects on other indicators. The effects of additives were highly significant (*p* < 0.01) for all indicators except NH_3_-N/TN. However, only LA and AA content were significantly affected by both interactions (*p* < 0.01). At two moisture content gradients, pH was significantly lower in both the LP and PA groups than in the CK group (*p* < 0.05). LA was significantly higher in the AhGLP and AhGPA than in the AhGCK (*p* < 0.05). PA was significantly lower in the AhGLP group than in other treatments (*p* < 0.05), and it was also significantly lower in the AhSCK and AhSLP than in AhSPA (*p* < 0.05).

**Figure 1:**
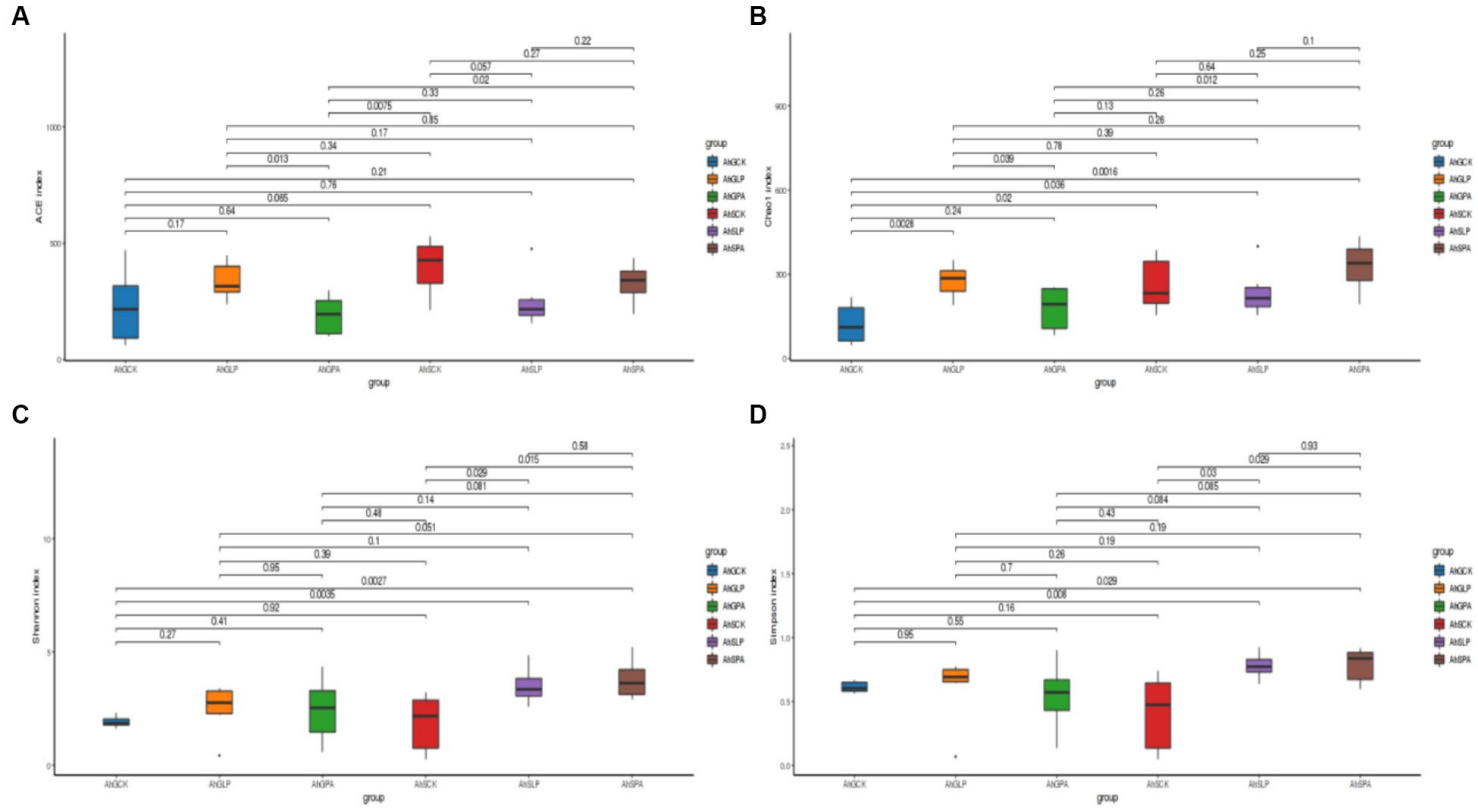
Observations on bacterial communities in amaranth silage bacterial species. **(A)** ACE index. **(B)** Chao1 index. **(C)** Shannon index. **(D)** Simpson index. AhG, 80% moisture content, AhS, 70% moisture content, CK, silage without inoculant; LP, inoculated with *L.plantarum*; PA, propionic acid.

Alpha diversity reflected the species abundance and diversity of individual samples (Figure 1). There was a significant difference in the Ace indices of AhGLP and AhGPA (*p* < 0.05), and highly significant difference in AhGPA and AhSPA (*p* < 0. 01). The treatment that differed in Chao1 index were AhGCK and AhGLP, AhGCK and AhSCK, AhGPA and AhSPA (*p* < 0.01). There were highly significant differences (*p* < 0.01) in the Shannon and Simpson indices for AhGPA and AhSPA, AhSCK and AhSLP, and AhSCK and AhSPA.

**Figure 2:**
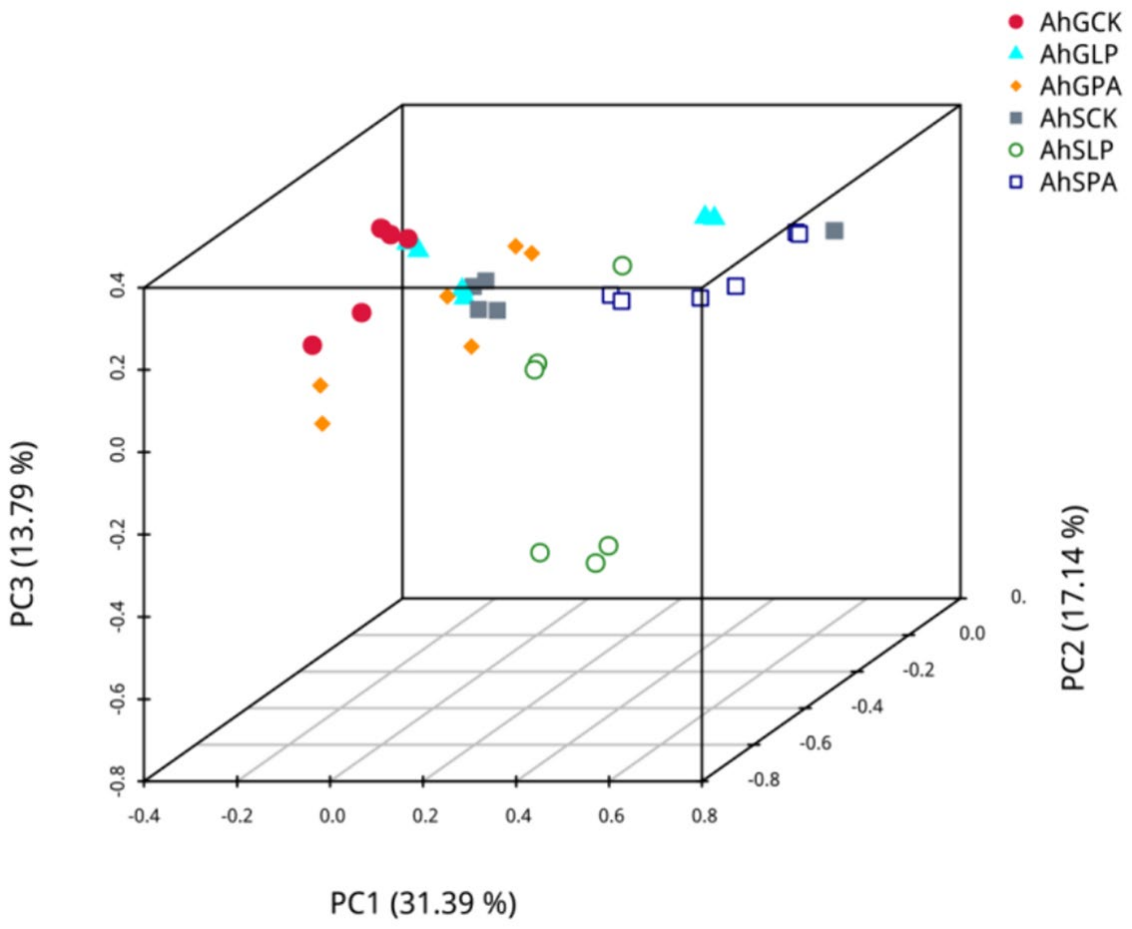
PCA plot based on OTUs of bacterial communities. The *X*-axis represents the first principal component, the *Y*-axis represents the second principal component, the *Z*-axis represents the third principal component, and the axis percentage represents the percentage contribution of the principal component to the sample variation. Each point in the graph represents a sample, samples in the same group are represented by the same colour, and samples in different subgroups are represented by different colours. AhG, 80% moisture content, AhS, 70% moisture content, CK, silage without inoculant; LP, inoculated with *L.plantarum*; PA, propionic acid.

PCA based on pearson’s distance was carried out to determine if there were differences in microbial community structure in the different silages (Figure 2). The PCA plots showed a clear separation of the bacterial communities of AhGCK, AhSLP and AhSPA from other treatments.

**Figure 3:**
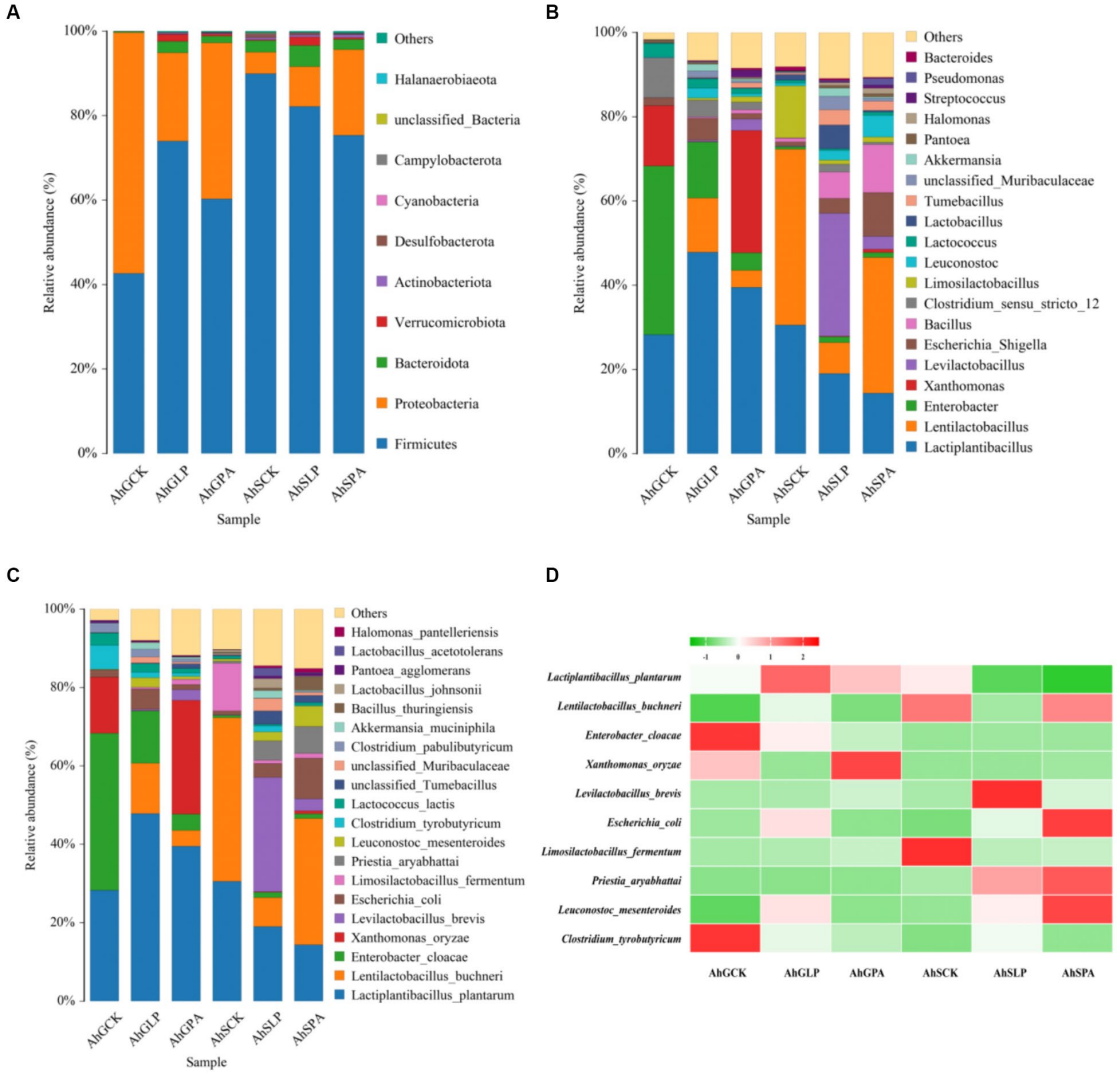
Bacterial community structure of amaranth silage before and after wilting. **(A)** Bacterial communities are shown at the phylum level (Top 10). **(B)** Bacterial community at genus level (Top 20). **(C)** Bacterial communities are shown at the species level (Top 20). **(D)** Heat map of changes in relative abundance of dominant species (Top 10) in the six treatments. AhG, 80% moisture content; AhS, 70% moisture content; CK, uninoculated silage; LP, *L. plantarum* inoculated; PA, propionic acid.

After 60 d of silage, the dominant phylum in the different treatments was mainly Firmicutes and Proteobacteria (Figure 3A). It is noteworthy that the relative abundance of Bacteroidota was high in all treatments except AhGCK. Meanwhile, the relative abundance of Verruomicrobiota in AhGLP and AhSLP was higher than other treatments. The dominant genus for AhGCK was *Enterobacter*, whereas the dominant genus of both AhGLP and AhGPA was *Lactiplantibacillus* (Figure 3B). The dominant genus for both AhGCK and AhGPA was *Lentilactobacillus*, and the genus with the highest relative abundance for AhSLP was *Levilactobacillus*. In particular, undesirable *Xanthomonas* were also present in AhGCK and AhGPA and were proportionally high in relative abundance. The dominant species of AhGCK in high-moisture silage was *Enterobacter cloacae*, followed by *L. plantarum* (Figure 3C). After the addition of *L. plantarum* and PA, the relative abundance of *Enterobacter cloacae* decreased and *L. plantarum* and *Lentilactobacillus buchneri* increased, but the high relative abundance of *Xanthomonas oryzae* (*X. oryzae*) was also present in AhGCK and AhGPA, and the relative abundance of *X. oryzae* increased instead after the addition of PA. In low-moisture silage, the dominant species for AhSCK and AhSPA was *Lentilactobacillus buchneri*, while the dominant species for AhSLP was *Levilactobacillus brevis*. The relative abundance of *Levilactobacillus brevis* and *Enterobacter cloacae* increased with the addition of *L. plantarum* and PA, in contrast to a decrease in the relative abundance of *L. plantarum* and *Lentilactobacillus buchneri*. A heat map of species abundance using the top 10 ranked species common to the six treatments revealed that some unfavourable fermentation species (*Enterobacter cloacae*, *X. oryzae*, *Clostridium tyrobutyricum*, etc.) were found to have high relative abundance in high-moisture silage (Figure 3D).

**Figure 4:**
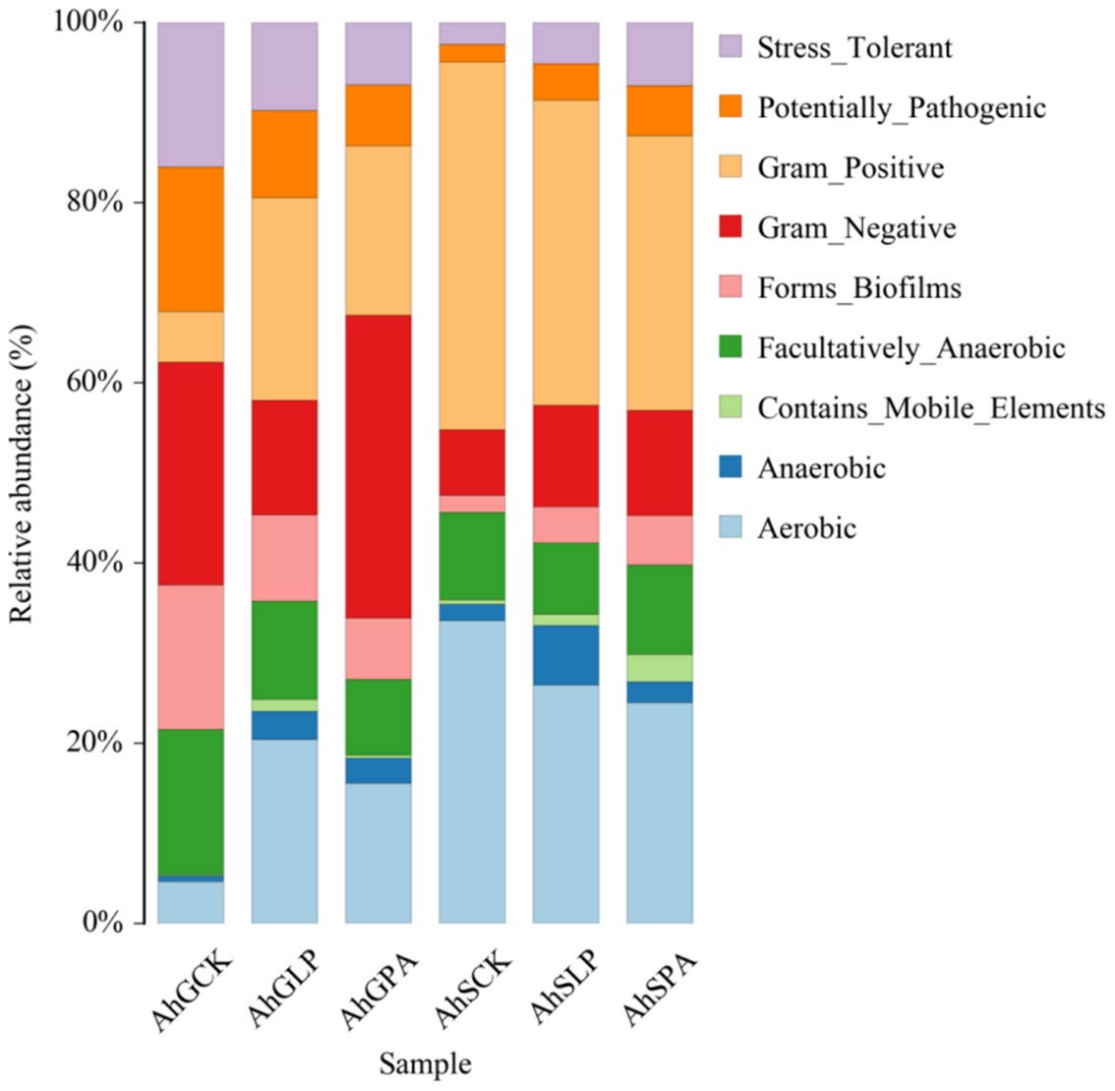
Predicted bacterial community phenotypes of different amaranth silage under different moisture gradients. AhG, 80% moisture content; AhS, 70% moisture content; CK, uninoculated silage; LP, *L. plantarum* inoculated; PA, propionic acid.

Predictive analyses of different silage bacterial community phenotypes were performed based on BugBase analysis (Figure 4). The proportions of aerobic bacteria, anaerobic and gram-positive bacteria were significantly higher (*p* < 0.05) in high-moisture silage samples for silage containing additives. However, the proportions of facultatively-anaerobic bacteria, potentially-pathogenic bacteria, and stress-tolerent bacteria were significantly reduced (*p* < 0.05). AhGCK and AhGPA had the highest proportion of gram-negative bacteria (*p* < 0.05), and AhGLP had the highest proportion of gram-positive bacteria (*p* < 0.05). The proportion of aerobic bacteria was significantly reduced (*p* < 0.05) in low-moisture silage samples for silage containing additives. However, the other bacterial communities were increased to different degrees (*p* < 0.05). The bacterium with the highest percentage in the three treatments with low moisture was gram positive.

**Figure 5:**
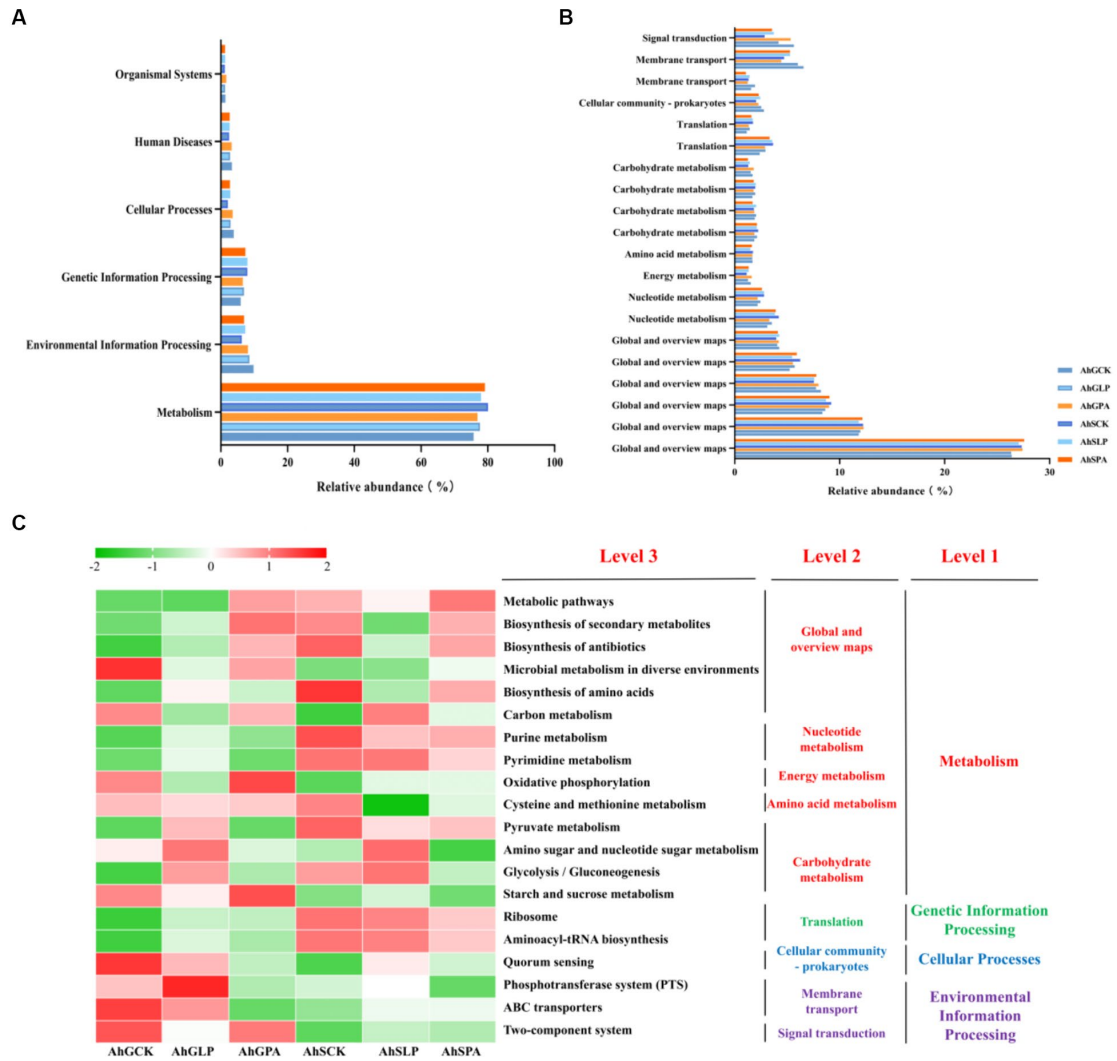
Functional predictions for silage microbiota with significantly different KEGG pathways (*p* < 0.05) among the six groups of silage (AhSCK, AhSLP, AhGPA, AhSCK, AhSLP and AhSPA). KEGG pathways at Level 1 **(A)**, Level 2 **(B)**, and Level 3 **(C)** are represented. AhG, 80% moisture content; AhS, 70% moisture content; CK, uninoculated silage; LP, *L. plantarum* inoculated; PA, propionic acid.

The bacterial metabolic functions were performed by the PICRUSt according to the KEGG pathway. A total of six different metabolic pathways, which were all primary metabolisms, emerged from the six treatments (Figure 5A). Metabolism was the main metabolic pathway, accounting for about 70%, followed by environmental information processing and genetic information processing. As shown in Figure 5B, which showed the top 20 metabolic functions in level 2, a total of 14 metabolic pathways were assigned to metabolism, and the remaining 6 pathways were assigned to genetic information processing, cellular process, and environmental information processing, respectively. Among the top 20 metabolic functions in tertiary metabolic pathways, six metabolic pathways (metabolic pathways, biosynthesis of secondary metabolites, biosynthesis of antibiotics, microbial metabolism in diverse environments, biosynthesis of amino acids, and carbon metabolism) were assigned to global and overview maps. It was followed by carbohydrate metabolism, where a total of four metabolic pathways were annotated in this pathway (Figure 5C). There were two metabolic pathways annotated in nucleotide metabolism. Amino acid metabolism had only one metabolic pathway annotated.

## Discussion

The epiphytic microbiota of fresh forage fed to livestock is diverse and difficult to predict. Although epiphytic LAB of fresh forage is essential for natural silage fermentation (Pahlow et al., 2003), there are several other epiphytic microorganisms that have hidden dangers for silage fermentation, leading to undesired spoilage of fermentation quality. In the present study, there were no differences in the nutrient composition of ingredients with different DM contents, but it should be noted that none of the attached LAB reached the standard of high-quality silage (>10^5^ lg cfu/g FM) and WSC content used as fermentation substrate was below 60 g/kg DM (Zhang et al., 2016). Fresh amaranth did not meet this criterion. However, the levels of *Escherichia coli*, yeasts and molds in FM were lower than the levels of harmful microbial bacteria found on *Oryza sativa*, *Leymus chinensis* and *Cyperus papyrus* in previous studies (He et al., 2020; Wu et al., 2022). Therefore, silage additives are considered to be indispensable additives in the silage preparation process.

After 60 d of fermentation, the CP content of AhGCK was significantly lower than AhSCK, which may be related to the biological activities such as reproduction of different microorganisms, harmful or beneficial, in the silage material. The osmotic pressure in the cells of the silage material kept increasing under low moisture content conditions, thus effectively inhibiting the activity of harmful microorganisms that can decompose proteins and various types of nutrients, and decreasing the production of BA and NH_3_-N (Xie et al., 2021), and CP content of *L. plantarum*-added silage showed the same trend as the control at different moisture content gradients. In general, the CP content in silage shows a decreasing trend as the anaerobic fermentation process advances, and the biological activity of adherent microorganisms affects the degree of degradation of CP content after the addition of exogenous additives (He et al., 2018). Interestingly, it was found that CP content increased in high-moisture silage with the addition of PA, which could be due to the addition of PA inhibited the hydrolysis of phytoproteinases and microbial protein production by undesirable microorganisms, such as *Clostridium* and *Enterobacter* (Wang et al., 2019), and avoided nutrient loss during silage process. There is also a possible reason that the additives effectively reduced pH of the silage, thus suppressed undesirable microorganisms growth and activity (Arriola et al., 2011). The findings of this study are in agreement with He et al. (2020) and Kaewpila et al. (2020).

The pH of all treatments (except for the AhGCK and AhSCK) after 60 d of ensiling, dropped below 4.20, which is an important indicator that the silage is well preserved (Webster, 1992). Compared to the control, the pH of silage with additives decreased significantly. High pH of AhGCK and AhSCK (5.09 and 4.51, respectively) indicated that the production of alkaline substances such as ammonia nitrogen due to protein hydrolysis during silage is more abundant (He et al., 2020). In addition, LAB attached to the surface of the forage are a key indicator of silage fermentation. In the present study, LAB counts of AhGCK and AhSCK did not reach 10^5^ lg cfu/g FM, suggesting that direct silage of amaranth was not easily successful. By the action of microorganisms, WSC are depleted and degraded into organic acids. However, amaranth did not have a high WSC content, and the low number of LAB attached to the raw material does not allow for the production of sufficient LA to lower the pH of the silage (Ma et al., 2023), which is compatible with the results of high pH and low LA content in the present study. In addition, inoculation with *L. plantarum* promoted homofermentation and prevents organic degradation caused by insufficient LA production (Ren et al., 2020), which is conducive to reducing nutrient losses. In this study, AhGLP, AhSLP, and AhSPA were found to have pH ≤ 4.20, a result that aptly confirms the effectiveness of *L. plantarum* in amaranth silage with different moisture contents.

Fermentation is a complex microbial symbiotic system, and microbial diversity influences fermentation quality and nutrient composition (Zhou et al., 2016). The microbial community of amaranth silage with two moisture content gradients containing additives were analysed by 16S high-throughput sequencing as a means of investigating the effects of moisture content and additives on the diversity of the microbial community. Microbial composition, species and numbers affect silage fermentation, and reduction of pH during ensiling inhibits microbial activity, leading to a reduction in microbial diversity (Feng et al., 2022). Allen et al. (2009) stated that low abundance of dominant flora and high species diversity in silage can lead to fermentation failure (Ni et al., 2018). The Shannon and Simpson indices of AhSCK were significantly different from both AhSLP and AhSPA, indicating that the addition of *L. plantarum* and PA to amaranth silage with low-moisture content reduced species diversity, suggesting that exogenous silage additives inhibited the growth and multiplication of harmful bacteria in the silage and dominated the whole fermentation process with *Lactobacillus* as the dominant bacterium, which is consistent with the findings of Wang et al. (2019) and Yang et al. (2019). It was possible to show the differences in bacterial communities between different silages at two moisture content gradients by PCA analysis, with AhGCK, AhSLP and AhSPA being clearly separated from other treatments, as evidenced by the differences in the fermentation characteristics of different treatments.

The exogenous addition of *L. plantarum* and PA altered the bacterial community composition of amaranth silage at different moisture contents, but Firmicutes and Proteobacteria were always the dominant phylum, which was consistent with the results of Wang et al. (2021) and Wu et al. (2020). There were some differences in the bacteria dominating the fermentation in different treatment, but the common dominant strain among treatments was *L. plantarum*, which is a homozygous fermenting LAB in six treatments, which can increase the LA content of silage, reduce the pH rapidly, and avoid the decomposition of the nutrients in the silage by some harmful microorganisms intolerant of acid (Zhao et al., 2023), which indicated that *L. plantarum* has a certain promotion effect on fermentation (Stokes, 1992). In this study, in terms of dominant bacteria in naturally fermented high-moisture silage, *Enterobacter cloacae* occupied the main predominant position, and the relative abundance of *X. oryzae* was also present in AhGCK and AhGPA occupying a high proportion. It explains why the pH was high in both treatments. The reason was that *Enterobacter cloacae* have deamino and decarboxylation effects on amino acids during fermentation, increase the amount of ammonia and biogenic amines by reducing NO_3_^−^, and will also compete with LAB for nutrients during the fermentation process. However, their growth and reproduction decreased with decreasing pH (Queiroz et al., 2018), and it was clear that AhGCK was affected by the high relative abundance of *Enterobacter cloacae* and *X. oryzae* may lead to high pH and poor fermentation quality. *X. oryzae* is a group of gram-negative bacteria that are also plant pathogens that consume the host’s own nutrients. *X. oryzae* was not found in silage containing *L. plantarum*, which indicated that the addition of *L. plantarum* may have inhibited the growth activity of harmful bacteria in high-moisture amaranth silage, avoiding their immunity from the host and the acquisition of nutrients from the plant (Boch and Bonas, 2010). In addition, the relative abundance of *Enterobacter cloacae* was lower in all treatments except AhGCK, which may be related to the level of moisture content and exogenously added silage additives.

In AhSCK, AhSLP and AhSPA, *Lentilactobacillus buchneri* and *Levilactobacillus brevis* acted as dominant bacteria in silage and as heterozygous fermenting LAB that could produce both LA and AA. Although their acid production rate is slower compared with homofermentative LAB, they can inhibit the growth of harmful bacteria during the aerobic phase, improve the aerobic stability of silage and inhibit the production of secondary fermentation (Okoye et al., 2023). The combined action of *Lentilactobacillus buchneri* and *Levilactobacillus brevis*, and *L. plantarum* inhibited the vital activities of spoilage microorganisms, changed the structure of microbial communities, and improved the fermentation characteristics of low-moisture amaranth silage. During the silage process, *Lentilactobacillus buchneri* converts LA to AA and 1,2-propanediol, effectively inhibiting the growth of molds and yeasts and improving aerobic stability (Xu et al., 2019). In this study, the relative abundance of *Lentilactobacillus buchneri* was higher in AhSCK and AhSPA than in the other treatments and the AA content was also high, but lower than in AhSLP. *Levilactobacillus brevis* is a heterotypic LA fermenting bacteria, compared with homotypic fermenting LAB, although its acid production rate is slower, but its products such as LA and AA can inhibit the growth of harmful bacteria, improve the aerobic stability of silage, and inhibit the production of secondary fermentation, thus improving the quality of fermentation (Ranjit and Kung, 2000). The relative abundance of *Levilactobacillus brevis* in AhSLP was higher than the other treatments, resulting in high LA content and low pH in this treatment, which in turn inhibited the growth and multiplication of spoilage microorganisms.

In this study, high relative abundance of *Enterobacter cloacae* and *Clostridium tyrobutyricum* was detected only in AhGCK, and the results of this study corroborate that *Enterobacter cloacae* and *Clostridium tyrobutyricum* slowed down the decrease of pH, competed with LAB for the fermentation substrate, and reduced the production of organic acids, decreasing the quality of silage fermentation (Pan et al., 2021). *X. oryzae*, as a pathogenic bacterium, occupies a large proportion of AhGCK and AhGPA, which may be one of the reasons for its poor silage quality.

We have used an algorithm called BugBase, which uses genome-wide birdshot or marker gene sequencing data to predict biologically interpretable phenotypes in complex microbiomes such as oxygen tolerance, gram staining, and pathogenic potential (Liu et al., 2023). In comparison with low-moisture silage, high-moisture silage samples had reduced proportions of gram-negative bacteria, potentially pathogenic bacteria and facultatively-anaerobic bacteria, and varying proportions of gram-positive bacteria, aerobic bacteria and anaerobic bacteria. During silage fermentation, the anaerobic and acidic environment makes the life activities of undesirable microorganisms, including potentially pathogenic bacteria, inhibited. Gram-negative bacteria and gram-positive bacteria multiply rapidly and become dominant, which dominate the whole fermentation process, thus leading to a decrease in microbial diversity, and microbial community structure completes a dynamic succession from gram-negative bacteria to gram-positive bacteria (Du et al., 2023). This indirectly explains the high quality of low-moisture silage and is consistent with the results of the species composition of the bacterial community in each treatment. Thus, the BugBase microbial community phenotype prediction analyses help to enhance the overall understanding of microbial information in silage microcosms.

**Figure 6:**
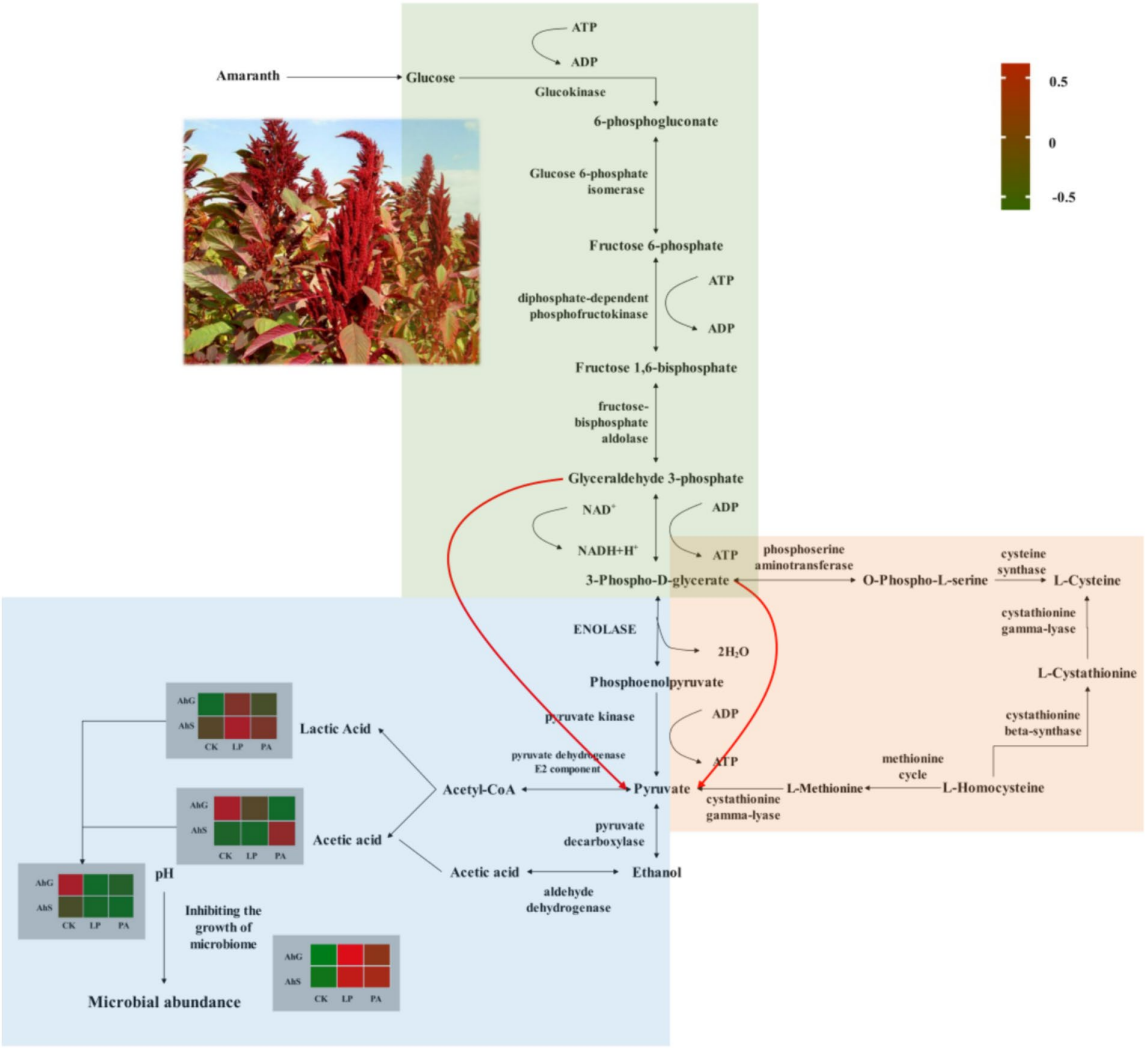
Overview of fermentative metabolic pathways in amaranth. Green background indicated glycolytic metabolism, orange background indicated cysteine and methionine metabolism, and blue background indicated pyruvate metabolism. AhG, 80% moisture content; AhS, 70% moisture content; CK, uninoculated silage; LP, *L. plantarum* inoculated; PA, propionic acid.

It has been suggested that inoculation additives can modulate microbial community dynamics and functional transfer during the silage process (Bai et al., 2021; Xu et al., 2021). The prediction of bacterial community function enabled us to assess the influence of bacterial communities on changes in silage metabolic pathways during ensiling (Guo et al., 2023). Amino acid metabolism, nucleotide metabolism, carbohydrate metabolism, cofactor and vitamin metabolism and energy metabolism are the main metabolic pathways during ensiling (Bai et al., 2021; Hisham et al., 2022; Wang et al., 2022), and we have analysed the effects of different additives on the main metabolic pathways of silage at different moisture content gradients using the PICRUSt2 bacterial prediction function. The relative abundance of amino acid metabolism was lower in AhSLP and AhSPA than in other treatments, which was related to the low pH of AhSLP and AhSPA, which was consistent with previous findings (Yuan et al., 2020). The higher abundance of carbohydrate metabolism in AhGLP, AhSLP and AhSPA than in other treatments was attributed to the fact that the dominant flora in the silage enhanced the fermentation process by up-regulating the carbohydrate metabolism (Xu et al., 2021; Bai et al., 2022). The AhGLP, AhSLP, and AhSPA groups had lower pH and higher LA concentrations compared to AhGCK, AhGPA, and AhSCK, whereas the AhGCK, AhGPA, and AhSCK had higher AA and PA concentrations than the AhGLP, AhSLP, and AhSPA. These differences may be attributed to the higher relative abundance of *X. oryzae* in AhGCK and AhGPA and lower abundance of pyruvate metabolism (Figure 6). The relative abundance of nucleotide metabolism was higher in low-moisture silage than in high-moisture silage, and nucleotides can be used to synthesise DNA and provide the main energy for cellular processes (Kilstrup et al., 2005; Wang et al., 2022). Interestingly, the changes in nucleotide metabolism in this study were opposite to the changes in energy metabolism. Therefore, other histological approaches such as proteomics, macrogenomics and metabolomics are needed to further elucidate the functional annotations and metabolic pathways of the microbial communities of silage with different moisture contents.

## Conclusion

The addition of PA was effective in increasing the CP content at high moisture content compared to *L. plantarum*, but the high moisture content promoted the growth of *X. oryzae*. The addition of *L. plantarum* reduced the pH and increased the LA content of silage with two moisture content gradients, resulting in inhibition of harmful bacterial activity and an increase in the relative abundance of *Lentilactobacillus buchneri*, *Levilactobacillus brevis* and *L. plantarum*. *L. plantarum* and PA act synergistically after anaerobic fermentation to accelerate the succession of the dominant species from gram-negative to gram-positive bacteria, forming a symbiotic microbial network centred on LAB. Both wilting and additive silage preparation methods increased the degree of dominance of global and overview maps and carbohydrate metabolism and decreased the degree of dominance of amino acid metabolism categories. In conclusion, the addition of *L. plantarum* to silage can effectively improve the fermentation characteristics of amaranth, increase the diversity of bacterial communities, and regulate the microbial community and its functional metabolic pathways to achieve the desired fermentation effect.

## Data availability statement

The data presented in the study are deposited in the NCBI repository, accession number PRJNA1049970.

## Author contributions

MZ: Writing – original draft, Conceptualization, Formal analysis, Investigation, Methodology, Visualization. JB: Conceptualization, Formal analysis, Investigation, Writing – original draft. ZW: Supervision, Writing – review & editing. PS: Conceptualization, Formal analysis, Investigation, Writing – original draft. JL: Conceptualization, Formal analysis, Investigation, Writing – original draft. YY: Conceptualization, Formal analysis, Investigation, Writing – original draft. GG: Conceptualization, Funding acquisition, Investigation, Methodology, Validation, Writing – review & editing.

## Footnotes

^1^https://international.biocloud.net
