# Erratum: Utilisation of *Lactiplantibacillus plantarum* and propionic acid to improve silage quality of amaranth before and after wilting: fermentation quality, microbial communities, and their metabolic pathway

**DOI:** 10.3389/fmicb.2024.1469591

**Published:** 2024-08-07

**Authors:** Frontiers Production Office

**Affiliations:** Frontiers Media SA, Lausanne, Switzerland

**Keywords:** amaranth silage, fermentation quality, *Lactiplantibacillus plantarum*, microbial community, moisture content, propionic acid

Due to a production error, authors Muqier Zhao and Jian Bao were not correctly listed as shared first authors.

The publisher apologizes for this mistake. The original article has been updated.

